# New approaches for the quantification and targeting of noradrenergic dysfunction in Alzheimer's disease

**DOI:** 10.1002/acn3.51539

**Published:** 2022-03-15

**Authors:** Michael David, Paresh A. Malhotra

**Affiliations:** ^1^ Imperial College London and the University of Surrey UK Dementia Research Institute Care Research and Technology Centre Sir Michael Uren Hub, 86 Wood Lane London W12 0BZ UK; ^2^ Imperial College London, Brain Sciences South Kensington London SW7 2AZ UK; ^3^ Imperial College Healthcare NHS Trust, Clinical Neurosciences Charing Cross Hospital London W2 1NY UK

## Abstract

There is clear, early noradrenergic dysfunction in Alzheimer's disease. This is likely secondary to pathological tau deposition in the locus coeruleus, the pontine nucleus that produces and releases noradrenaline, prior to involvement of cortical brain regions. Disruption of noradrenergic pathways affects cognition, especially attention, impacting memory and broader functioning. Additionally, it leads to autonomic and neuropsychiatric symptoms. Despite the strong evidence of noradrenergic involvement in Alzheimer's, there are no clear trial data supporting the clinical use of any noradrenergic treatments. Several approaches have been tried, including proof‐of‐principle studies and (mostly small scale) randomised controlled trials. Treatments have included pharmacotherapies as well as stimulation. The lack of clear positive findings is likely secondary to limitations in gauging locus coeruleus integrity and dysfunction at an individual level. However, the recent development of several novel biomarkers holds potential and should allow quantification of dysfunction. This may then inform inclusion criteria and stratification for future trials. Imaging approaches have improved greatly following the development of neuromelanin‐sensitive sequences, enabling the use of structural MRI to estimate locus coeruleus integrity. Additionally, functional MRI scanning has the potential to quantify network dysfunction. As well as neuroimaging, EEG, fluid biomarkers and pupillometry techniques may prove useful in assessing noradrenergic tone. Here, we review the development of these biomarkers and how they might augment clinical studies, particularly randomised trials, through identification of patients most likely to benefit from treatment. We outline the biomarkers with most potential, and how they may transform symptomatic therapy for people living with Alzheimer's disease.

## Introduction

The mainstay of medical treatment for Alzheimer's disease (AD) for over two decades has consisted of pharmacological therapies targeting well‐established cholinergic deficits.^1^ In recent years development of new treatments has focussed on disease modification rather than the modulation of symptoms through targeting of dysfunctional neurotransmitter pathways. However, in addition to cholinergic disruption, there is clear and early pathological involvement of noradrenergic pathways in AD, with increasing evidence to suggest that this may be related to cognitive and behavioural dysfunction,^2^, ^3^, ^4^, ^5^, ^6^ and possibly even disease progression.^7^

Noradrenergic compounds have previously been trialled in dementia and AD in particular, but most of these studies were carried out in an era preceding the standardisation of randomised controlled trials and systematic AD diagnosis.^8^, ^9^, ^10^ Thus, trials tended to be underpowered and were extremely likely to have included patients with cognitive impairment secondary to other diseases.^11^ Within the context of biomarker‐confirmed AD, it is becoming increasingly clear that AD pathology and symptom profile vary between individuals and over time.^12^, ^13^ AD can present with a spectrum of cognitive impairments^14^—likely underpinned by varying involvement of different neurotransmitter systems. Hence future trials of noradrenergic therapies may benefit considerably from incorporating indices of neurotransmitter pathway‐specific dysfunction.

Until recently, noradrenergic involvement in AD could only be reliably measured at autopsy, limiting any possibility of biomarker‐informed treatment. This is in contrast to other neurotransmitters and causes of neurodegeneration, particularly dopamine and idiopathic Parkinson's disease (PD) and Dementia with Lewy Bodies.^15^ However, recent developments in imaging techniques^16^ and other physiological biomarkers such as EEG and pupillometry^17^, ^18^, ^19^ that enable the detection and measurement of noradrenergic dysfunction, highlight promising avenues for more targeted treatment. Below we outline the evidence for noradrenergic dysfunction in AD before reviewing the methods through which this can be identified and quantified to guide noradrenergic therapy.

## The locus coeruleus‐norepinephrine system

### Normal function

Noradrenaline (or norepinephrine (NE)) is a catecholamine synthesised and then released by noradrenergic neurons before acting on one of the three adrenoreceptor (AR) subtypes: α_1_, α_2_ and β.^20^

**Figure 1:**
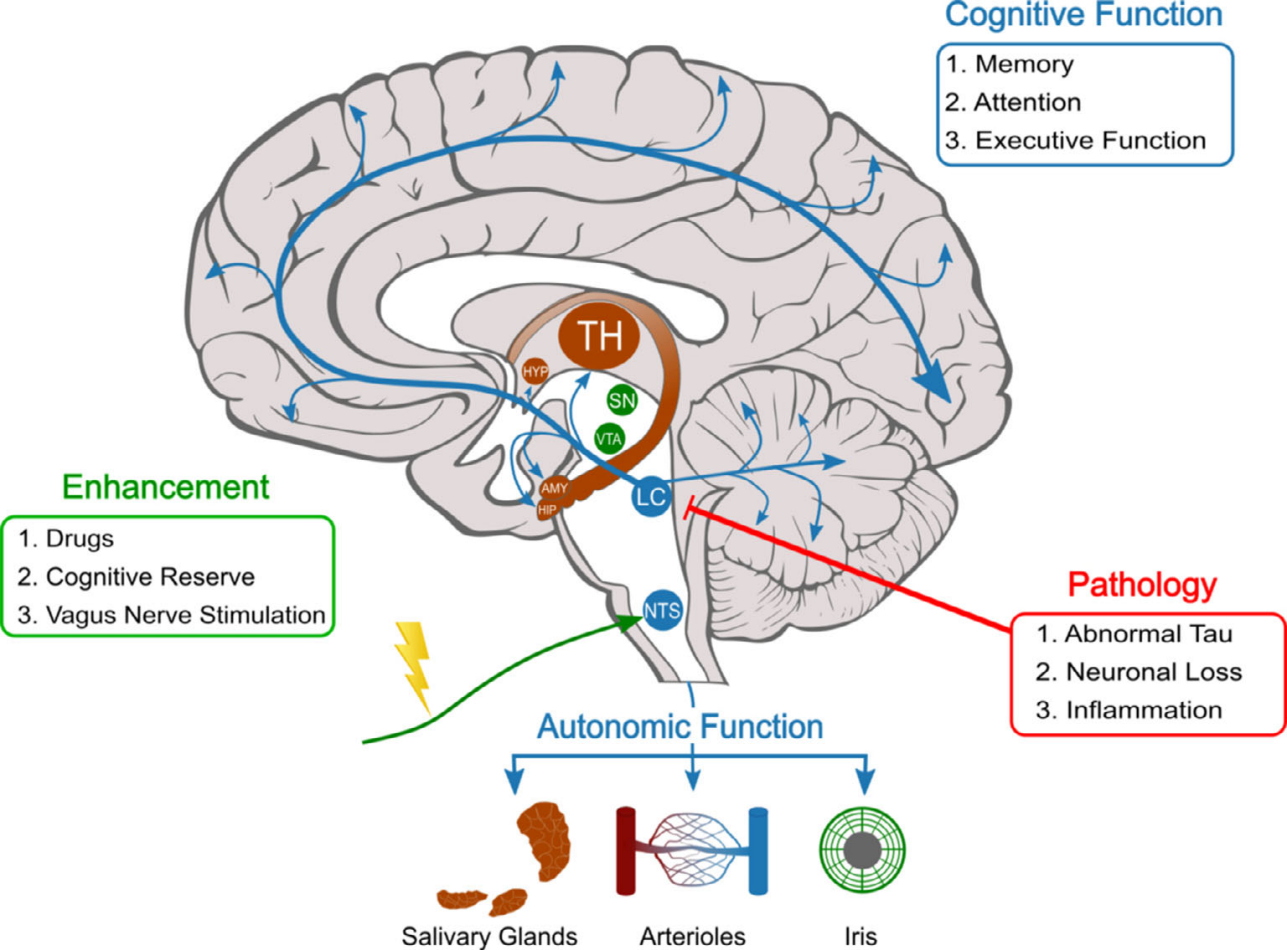
Schematic showing the locus coeruleus and its projections throughout the brain. Details of autonomic functions of the noradrenergic system are also shown. Actions of the noradrenergic system on cognition, as well as the nature of pathology seen in AD, and potential tools to modulate this system when dysfunctional are also shown. TH, thalamus; HYP, hypothalamus; SN, substantia nigra; VTA, ventral tegmental area; AMY, amygdala; HIP, hippocampus; LC, locus coeruleus; NTS, nucleus tractus solitarius.

The principal source of NE in the brain is the locus coeruleus (LC),^21^ which comprises a pair of tiny nuclei in the pons, from which cells project throughout the brain.^22^ As well as the thalamus, amygdala and hippocampus, noradrenergic neurons connect the LC with the entorhinal and frontal cortices,^23^ with extensive prefrontal cortex (PFC) inputs.^22^, ^24^, ^25^ In addition to general arousal and alertness,^26^, ^27^ they have been shown to influence attention,^28^, ^29^, ^30^ memory^31^, ^32^, ^33^ and executive function^3^, ^34^ (Fig. 1).

Recent understanding of the LC points to a modular structure with sub‐domains that project to terminal structures independently and as such have differential influence on behavioural and cognitive function.^35^, ^36^ It is increasingly possible to investigate and quantify the integrity of this system with a greater degree of precision with the development of modern biomarker techniques such as ultra‐high field 7‐Tesla magnetic resonance imaging (MRI).^37^

**Figure 2:**
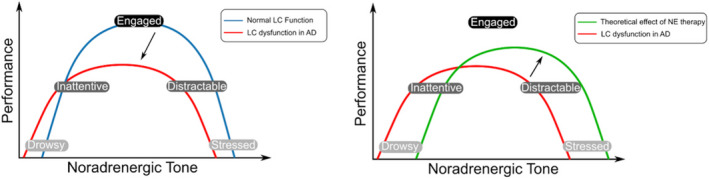
Schematic showing the Yerkes–Dodson arousal curve and the possible effects of noradrenergic therapy. (A) Shows the two theoretical versions of the arousal curve; one representing normal LC function in health (blue), and one representing the curve that may be seen with LC dysfunction in AD (red). The black arrow represents the transformation between the two curves. Pathology in this system in AD is likely to affect the dynamics of the ascending arousal system and disturb the balance of arousal seen in health. This dysfunction results in the curve moving to the left, representing a decreased likelihood that they will be in an ‘engaged’ state, whilst they also have an increased tendency to be in low performance states; ‘drowsy’ and ‘stressed’. (B) Shows the same curve for LC dysfunction in AD as well as the theoretical curve seen patients who have received noradrenergic therapy. The black arrow represents the transformation between the two curves. Noradrenergic therapy is theorised to heighten the noradrenergic tone in patients, moving the curve to the right, and therefore leading to an increasing likelihood that patients will be in an ‘engaged’ state. Successful use of noradrenergic treatments may rely on dose titration in order to ‘tune’ the system to the central peak of the classic inverted‐U curve.^136^ It has the potential to lead to ‘over‐arousal’, however, with the line shifted too far to the right, and therefore patients may be more ‘stressed’ than ‘engaged’. LC, locus coeruleus; AD, Alzheimer's disease, NE, noradrenaline.

According to the influential ‘adaptive‐gain theory’, the LC‐NE system has two modes of activity: tonic and phasic.^38^ Cognitive performance and phasic activity appear to be optimal at an intermediate level of tonic LC activity, in accordance with the classic Yerkes–Dodson arousal curve.^38^, ^39^, ^40^ Critically, this suggests that the relationship between LC‐NE dysfunction and any treatment response is not linear (Fig. 2).

### Noradrenergic dysfunction in Alzheimer's disease

In 1981, Tomlinson and colleagues discovered that AD leads to early neuronal loss in the LC^41^ and in the last 40 years, a range of functional and anatomical changes to the noradrenergic system have been observed. In fact, the LC is the site at which the accumulation of neurofibrillary tau tangles is first seen in AD, prior to their appearance in the medial temporal lobe.^42^ MRI sequences have more recently been developed to measure LC integrity^43^ and have corroborated evidence that early LC degeneration occurs in AD.^44^, ^45^ Critically, LC neuron loss^46^, ^47^, ^48^ and NE concentration in several brain regions^49^, ^50^ have been shown to correlate with severity of disease. A recent study from Jacobs et al. showed that MRI measures of LC integrity are associated with a pattern of tau accumulation that is consistent with Braak staging.^5^ They also showed that imaging measures of LC intensity are associated with cognitive decline in the context of elevated β‐amyloid in clinically normal older adults, with the authors proposing that these may be used as a predictor of AD‐related cognitive decline.^5^ Additionally, whilst it appears plasma and cerebrospinal fluid (CSF) levels of NE (and its metabolite 3‐methoxy‐4‐hydroxyphenylglycol (MHPG)) are maintained or even elevated in the earlier stages of AD, LC degeneration ultimately leads to decreased levels of NE in terminal regions.^49^

#### Symptoms associated with locus coeruleus‐noradrenaline dysfunction in Alzheimer's disease

##### Attentional deficits in Alzheimer's disease

Attentional functions have been examined systematically in AD^51^ and it is clear that attention is frequently affected early in the course of disease.^52^ In 1990, Posner and Peterson laid out their influential model for human attention, describing a combination of three interlinked networks; alerting, orienting and executive.^53^ Within the broad domain of attention, arousal and alerting appear to be especially reliant on an intact LC‐NE system.^3^ However, impairments of all three networks are likely to be caused by disruption of LC‐NE pathways as well as effects on other ascending arousal systems.^1^, ^3^ In their recent 7‐Tesla fMRI study, Munn and colleagues provided further evidence of how the LC‐NE system's role in ascending arousal system modulates the configuration of neural networks and subsequently influences conscious awareness.^54^ Deficits in awareness have consequences for patients' perceptual sensitivity^55^ and ability to orient attention to salient stimuli,^56^ with real‐world impact for patients. For example, in affecting their ability to drive on a busy road or to follow a conversation in a busy restaurant––situations in which orienting and sustaining attention are vital, and where patients with AD repeatedly report difficulties.

Attentional functions should not be considered in isolation from other cognitive domains in the context of noradrenergic dysfunction. Most relevant to AD, Clewett and colleagues revealed that the LC‐NE system influences the efficacy of memory formation under arousal.^57^ Using simultaneous functional MRI (fMRI) and pupillometry, they showed that greater scene encoding activity in the LC and parahippocampal cortex correlated with greater threat‐evoked pupil dilations. The LC engagement for encoding, as measured by pupil dilations, was correlated with neuromelanin signal intensity (a measure of LC integrity discussed in detail in Section 3.2.1.), providing evidence that LC structure relates to its activation pattern during cognitive processing.^57^ A further MRI study of healthy older adults showed participants with higher neuromelanin‐related signal intensity in the LC performed better on a memory task.^58^ This has clear implications for AD and suggests a mechanism by which impaired LC function might lead to impaired episodic memory.

##### Neuropsychiatric dysfunction

In addition to cognitive dysfunction, multiple neuropsychiatric symptoms are associated with AD, including depression, anxiety and psychosis.^59^ Much like the other symptoms described, these neuropsychiatric problems have a well‐established association with NE.^7^, ^60^ Patients with a history of depression have fewer LC neurons post‐mortem,^61^ and in dementia there is a positive correlation between LC degeneration and both depression^62^ and aggressive behaviour.^50^ As with cognitive function, the relationship between NE and neuropsychiatric state may not be linear. In fact, patients appear to be vulnerable to the effects of both over and under‐arousal. Thus there is a potential risk of inducing undesired states of stress with excessive noradrenergic treatment^63^ (Fig. 2).

##### Autonomic dysfunction

Autonomic dysfunction is common in AD but often overlooked, and LC‐NE disruption is implicated in its pathogenesis. Stimulation of the LC‐NE provokes a pressor response in the arterioles and increase in heart rate,^64^, ^65^, ^66^ as well as stimulation of the sweat glands, via sympathetic innervation.^67^ Symptoms such as orthostatic hypotension which, although more common in α‐synucleinopathies such as PD, are more prevalent in AD than in individuals without dementia.^68^ This may be through loss of LC projections to pre‐ganglionic neurons that increase sympathetic activity and decrease parasympathetic activity via the α_1_‐AR and α_2_‐AR, respectively.^67^ Pupillary fluctuations, under autonomic control, are related to arousal and can be measured as a proxy for LC function––as discussed in detail in section 3.3.

##### Sleep–wake cycle disturbance

Disturbance of the sleep–wake cycle is significantly more common in AD, often preceding cognitive symptoms.^69^ Symptoms include frequent awakenings, nocturnal wandering and excessive daytime sleepiness, together causing considerable burden to patients and their families. This may in part be due to tau accumulation in the LC which contains wake‐promoting neurons.^70^ This account is supported by correlation between symptoms of sleep disturbance and reduced LC integrity as measured by 7 T MRI.^71^

## Using biomarkers to guide therapeutic approaches

### Introduction

The NIH‐FDA Biomarker Working Group define a biomarker as a ‘characteristic that is measured as an indicator of normal biological processes, pathogenic processes, or responses to an exposure or intervention, including therapeutic interventions’.^72^ Here, we review the most promising biomarkers that investigators have proposed as indices of LC‐NE function, and the extent to which they satisfy that definition. In some instances, notably structural MRI, pupillometry and EEG, there are studies linking LC activity and integrity with biomarker changes in a causal manner, through the stimulation of blocking of LC activity, and we have referred to these accordingly. However, there is minimal evidence to‐date that functional MRI measures, for example, provide specific measurements of LC activity.

Neurodegenerative diseases lead to heterogeneous patterns of pathology and phenotypes.^12^ The extent of LC damage and noradrenergic dysfunction varies considerably across the AD spectrum. AD patients stratified to ‘early onset’ rather than ‘late onset’ show nearly four times greater likelihood of higher LC atrophy on autopsy after controlling for other measures of pathological progression. Although LC atrophy did not appear to affect overall cognitive performance in this study, it did correlate with attentional function.^47^

The effect of treatment may well also be influenced by disease progression. Dysregulation of the LC‐NE system in the early stages contributes to increased aberrant amyloid accumulation. Thus, using medication to modulate the accumulation of this pathology may be best used as the start of the disease process.^47^ Although the integrity of the LC and pharmacological responsiveness in prodromal AD need further investigation, this account would suggest that the stage of mild cognitive impairment (MCI) may offer a critical window of time to initiate novel noradrenergic‐based therapies.^7^

The difficulties associated with existing cholinergic treatments highlight the need for biomarkers to guide noradrenergic approaches if they are to be successful. Cholinergic effects are modest at the group level but the benefit to some individuals is perhaps underestimated. This may to some extent, be due to the insensitivity of the tools traditionally used to assess improvement but, regardless, there is considerable variability in response to cholinergic treatment between individuals, highlighting the need for tools to identify those most likely to respond.^74^ This approach has been developed in the context of methylphenidate both in attention deficit hyperactivity disorder (ADHD) where children who have particularly poor sustained attention are most likely to benefit^75^ and traumatic brain injury in which drug response has been linked to neuroimaging markers.^76^ There has also been preliminary success with a similar approach being used to correlate LC integrity and response to a single dose of noradrenergic treatment in PD.^77^

To this end, there is a current need to develop robust biomarkers of LC‐NE dysfunction to guide and inform trials, as well as confirm mechanisms of action and allow for targeted treatment. Such an approach would enable individualising of treatment in accordance with the indexed pathology, in contrast to findings from more broadly inclusive randomised controlled trials that might be misleading by averaging results across heterogeneous cohorts.^78^, ^79^ Furthermore, use of biomarkers that index noradrenergic activity could potentially provide important information regarding the therapeutic mechanism of interventions that act via multiple neurotransmitter systems.

### Imaging markers of the locus coeruleus‐noradrenaline system

Due to its small size, low magnetic resonance signal in conventional T1‐weighted MRI and location deep in the brainstem where it is susceptible to artefacts from adjacent blood vessels, the LC has historically been difficult to image.^37^, ^80^, ^81^ However, recent advances in structural and functional imaging techniques^82^ have furthered our understanding of the noradrenergic system and allowed evaluation of its integrity in ageing and disease.^16^, ^80^, ^83^, ^84^, ^85^ It is worth noting, however, that unlike Ioflupane SPECT in PD, there is currently no direct functional measure of LC‐NE integrity.

#### Structural

Over the last decade, researchers have imaged the LC more precisely using MRI sequences sensitive to neuromelanin, a paramagnetic compound that accumulates in noradrenergic neurons.^43^ Neuromelanin‐sensitive MRI constituted a major breakthrough in allowing accurate segmentation of the LC. Signal intensity (or contrast) on these sequences can be used as a measure of LC integrity.^85^, ^86^ A recent study showed the validity of this biomarker as an accurate measure of pathology by correlating MRI and post‐mortem findings.^5^

However, this technique is not without its limitations. Substantial interindividual differences in LC measures are seen across healthy older adults^16^, ^87^ and neuromelanin increases across the lifespan.^87^ Furthermore, precise understanding of this variation is lacking.^85^ We also note that neuromelanin‐sensitive MRI allows quantification of cell density, but not synaptic density, and not cell activity.^85^

**Figure 3:**
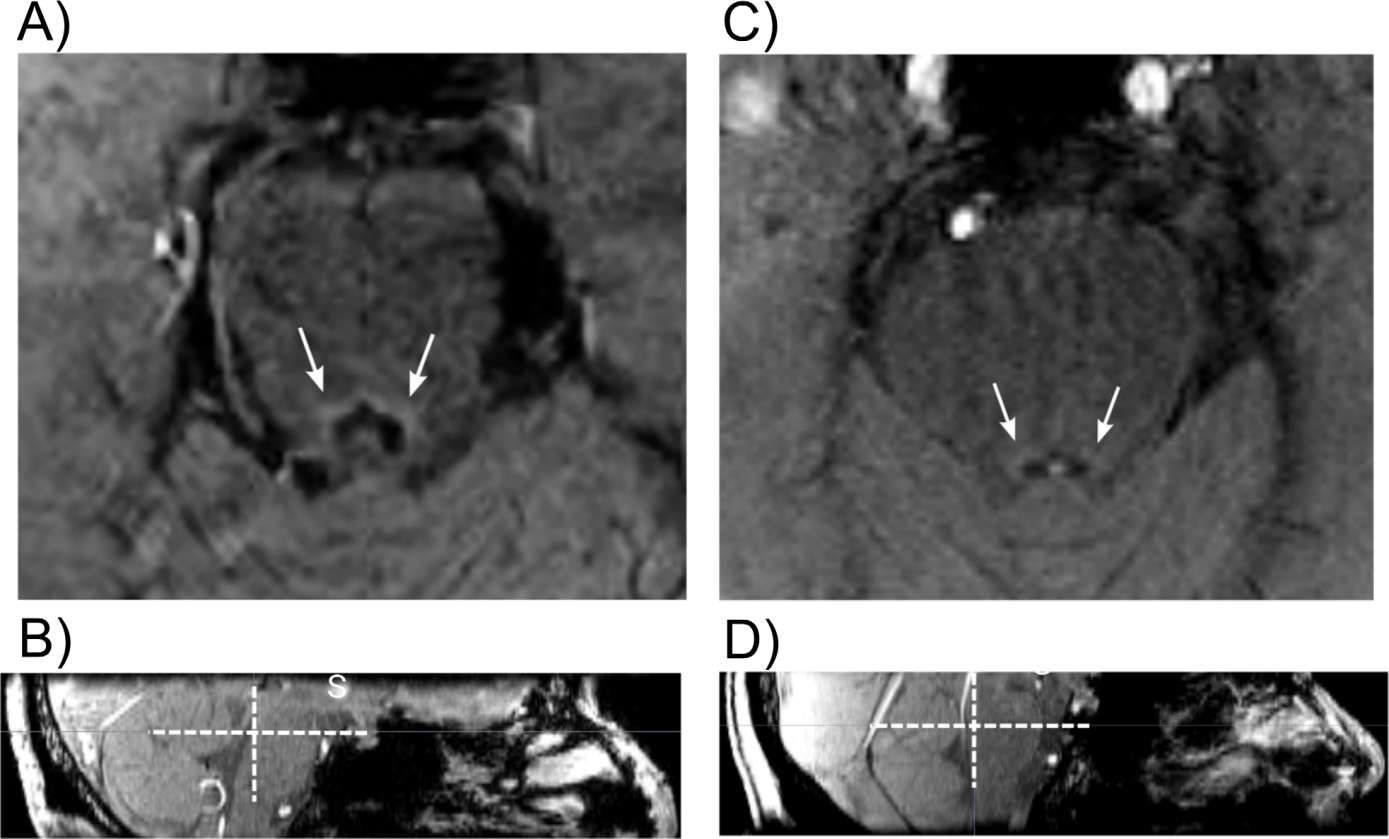
Single slices from a 3‐Tesla magnetization transfer contrast MRI of the pons by our group. (A) (Axial) and (B) (sagittal) are from a 62‐year‐old healthy control, showing a healthy LC with clear contrast compared to the rest of the pons. (C) (Axial) is (D) (sagittal) are from 71‐year‐old patient with Alzheimer's disease with reduced contrast at the site of the LC, showing evidence of degeneration. White arrows and crosses highlight locus coeruleus (LC).

As stated above, MRI has been used to differentiate AD patients from healthy controls, at the group level, based on LC integrity^44^, ^45^ (Fig. 3). Recent advances in structural LC imaging may provide the key to unlock the potential of noradrenergic treatments in AD by enabling the stratification of patients in clinical trials according to the degree of noradrenergic dysfunction.^85^ This may be improved further by higher resolution methods, and multiple studies have shown the LC can been successfully imaged using 7‐Tesla MRI.^88^, ^89^, ^90^ Work in PD has shown that individual differences in LC structure measured using neuromelanin sequences at 7‐Tesla correlate with response to noradrenergic modulation of cognitive impairment, suggesting that a similar approach may be possible in AD^79^. This single dose proof‐of‐principle study points the way for longitudinal, prospective studies of noradrenergic treatments with systematic evaluation of the correlation between individual response and imaging findings.

#### Functional

Resting state fMRI studies have shown functional connectivity (FC) between the LC and various brain regions^91^ in healthy humans, most convincingly the amygdala.^16^ With regards to memory, greater FC with the parahippocampal gyrus^92^ and cerebellum^93^ have been correlated with performance in people with early dementia and parental history of AD, respectively. In terms of FC changes seen in clinical AD, there are inconsistencies in the literature,^94^, ^95^ although there is evidence suggesting an important role for the LC. One study of AD patients demonstrated the LC to be one of the two foci (along with right orbitofrontal cortex) of disrupted paths of connectivity, when compared to controls.^96^ These findings were corroborated in a later study in which results reached significance when using more generous statistical methods to account for the limited imaging resolution.^97^ Interestingly, individuals with MCI have been found to have disrupted LC‐parahippocampal gyrus connectivity, whilst this measure still correlated with memory performance in higher functioning individuals, suggesting that altered FC is detectable early in the disease process.^92^

Taken together, there is significant evidence for the potential of fMRI as an adjunct to structural imaging in indexing LC‐NE dysfunction in this patient population. Whilst quantification of LC integrity with structural imaging can be used to index dysfunction on an individual level, functional neuroimaging has the potential to demonstrate treatment‐related changes in brain activity in tandem with established neuropsychological measures of cognitive response. Furthermore, given the relatively non‐specific action of many neuropsychiatric drugs, these techniques might also be used to assess the extent to which the noradrenergic action of the drug is contributing to said response. It is likely, however, that structural imaging will be implemented in the guidance of noradrenergic treatment some time before functional imaging can reliably be used in this way.

Positron emission tomography (PET) scanning uses radioligands to assess markers of brain function such as changes in metabolism and neurotransmitter levels.^86^ PET has poorer spatial resolution compared to MRI and it is also more expensive and invasive than MRI, but offers additional information, depending on the tracer used.^86^ For example, imaging of the intracellular NE transporter using PET has shown lower binding in LC and thalamus which correlated with post‐mortem Braak staging.^98^ Tau‐PET has revolutionised the understanding of Alzheimer's pathophysiology but this technique's resolution remains too low at present to reliably detect tau pathology in LC.^99^ Furthermore, subcortical Tau‐PET findings need to be interpreted with caution given off‐target binding to neuromelanin, which would likely be dependent on age as well as disease.^85^

### Pupillometry

There has been a considerable recent increase in the volume of research using pupil dynamics as a route to understanding brain function, and ascending arousal systems in particular.^100^ Baseline and evoked changes in pupil size are influenced by multiple brain regions and neurotransmitters, including dopamine, but there is compelling evidence directly linking noradrenergic pathways with pupil dilation.^101^ Work in mice has shown pupil dilation increases monotonically with the number of LC spikes, although the relationship is highly variable.^102^ More relevant to humans, a study by Joshi and colleagues in macaques, has demonstrated that stimulated LC activity leads to associated changes in pupil size.^101^ Therefore changes in pupil diameter may provide a reliable index of LC‐NE function^40^, ^103^, ^104^ and pupillometry holds promise as a biomarker of this system in AD.

Multiple pupillometry indices have been linked to tonic and phasic LC‐NE function in animals and healthy humans,^40^ and have shown to vary between AD/MCI patients and healthy controls.^17^ Pupillometry has also been used in conjunction with fMRI studies to evaluate the interplay between resting LC tone and brain activity during cognitive tasks.^101^, ^105^, ^106^ For example, as mentioned above, LC‐NE activity, as indexed using pupillometry, has been associated with success of memory encoding.^57^

Crucially, there is potential to use pupillometry to not only detect noradrenergic dysfunction and thus predict treatment response, but also monitor treatment effect. One study of the effect of methylphenidate, a norepinephrine–dopamine reuptake inhibitor, in ADHD patients showed that, when off treatment, they had a decreased pupil diameter during an attentional task compared to healthy controls. The difference between groups was not present when the patients were on noradrenergic treatment.^107^ Relative to neuroimaging, pupillometry is cheap, quick, portable, and well tolerated and thus has potential to be used at scale and longitudinally.

### Electroencephalogram

Event‐related potential (ERP) measured using electroencephalogram (EEG) correlates with pupillometry measures of LC function.^40^ Importantly, this technology has the potential to be used practically in outpatient settings.

As per pupillometry, EEG may be used to index LC activity and can potentially also be used to gauge the effect of noradrenergic treatment. Rodent studies using pharmacological infusions at the site of the LC showed EEG responses to activation, with a shift from low‐frequency, high‐amplitude to high‐frequency, low‐amplitude EEG activity in the frontal neocortex as well as the appearance of theta‐rhythm in the hippocampus,^108^ whilst a shift from high‐frequency, low‐amplitude to low‐frequency, high‐amplitude EEG activity was seen in response to *inactivation*.^26^

Cecchi and co‐workers identified ERP features associated with reduced attention, working memory, and executive function in an AD patient group.^18^ Further studies in AD patients have revealed decreases in gamma frequency bands,^19^ putatively related to attention, and memory storage and retrieval.^109^ The activation of gamma‐aminobutyric acid (GABA)‐A receptors is thought to promote these gamma oscillations^110^ and GABA‐induced inhibition can be augmented by NE.^111^ Therefore, a reduction in LC‐NE activity and NE levels could result in a decrease in GABAergic inhibitory activity leading to the abnormalities in gamma oscillations seen in AD.

Crucially, selective NE reuptake inhibitors have been shown to enhance gamma activity in rats,^112^ showing how EEG could be used to index noradrenergic treatment. However, equivalent studies have yet to be performed in humans.

Both EEG and pupillometry offer a way of assessing noradrenergic tone *in real time*. High temporal resolution in measurements of response to stimuli may allow for greater understanding of the underlying neural networks at play, especially when used in conjunction with pupillometry.^113^ Either of these media could guide treatment if used simultaneously with non‐pharmacological noradrenergic therapies, for example vagal nerve stimulation (VNS), which is known to affect noradrenergic pathways,^114^ (see Section 4.4).

### Polysomnography

Polysomnography is the gold standard technique for assessment of physiologic parameters during sleep.^115^ It has been successfully used in AD, showing that sleep efficiency and pattern is deleteriously affected when compared to controls.^116^, ^117^ There is growing evidence from this methodology of the relationship between sleep/wake disturbances and LC changes in AD.

As for treatments, current therapies used for sleep problems in AD are non‐specific, causing side effects (e.g. sedation, falls). The use of polysomnography provides the potential to target treatment for those patients with LC dysfunction that may be affecting the sleep–wake cycle.^118^ New technologies have enabled higher quality home assessment of sleep physiology, and these are likely to lead to better diagnosis of sleep abnormalities^119^ and targeted treatment approaches.

### Fluid biomarkers

Whilst LC degeneration ultimately leads to decreased levels of NE in terminal regions, there is evidence that plasma and CSF levels of NE (and its metabolite 3‐methoxy‐4‐hydroxyphenylglycol (MHPG)) are maintained or even elevated in the earlier stages of AD^49^. As the disease progresses, however, plasma NE falls in correlation with cognitive function.^120^

### Potential applications

Given the methodological advances described above, there is great potential in the use of biomarkers for guiding and optimising treatment in this context. Structural imaging can provide the opportunity to identify patients with damage to noradrenergic pathways. This can then be quantified and scaled in the broader context of the disease through techniques such as measuring the contrast ratio of the LC compared to surrounding reference regions.

**Figure 4:**
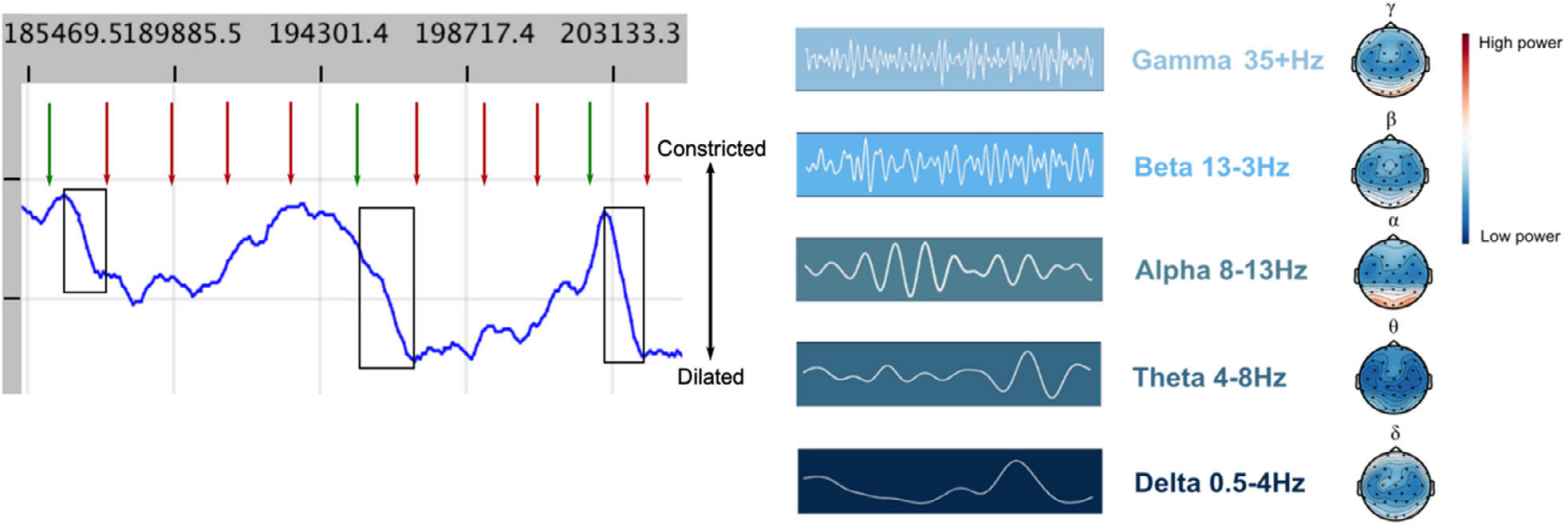
Examples of biomarker modalities that can be used to assess noradrenergic function in Alzheimer's disease. (A) Snapshot of unpublished pupillometry data from one individual during an oddball task. This task uses infrequent stimuli (oddballs), in a sequence of frequent stimuli to elicit a pupil dilation, that is used as a measure of attention as controlled by the LC‐NE system. Black box highlights pupil dilation in response to oddball (green arrow) rather than standard tone (red arrow). (B) Different frequency bands of EEG signal can be quantified. The highest frequency, gamma waves are thought to be altered in Alzheimer's disease as a result of noradrenergic dysfunction. Topoplots of unpublished healthy control data on the right of the figure show the different relative power across the cortex seen across the five frequency bands.

Using an experimental medicine approach, a relationship can then be established between results generated using biomarkers and the likelihood of a response to noradrenergic treatment. Prospectively, patients can be stratified based on a prediction of whether they are likely to respond, and hence treatment can be targeted at those most likely to benefit. This can also be enhanced through real‐time monitoring of the effect of treatment using modalities such as EEG and pupillometry (Fig. 4).

## Promising noradrenergic therapies

### Introduction

**Figure 5:**
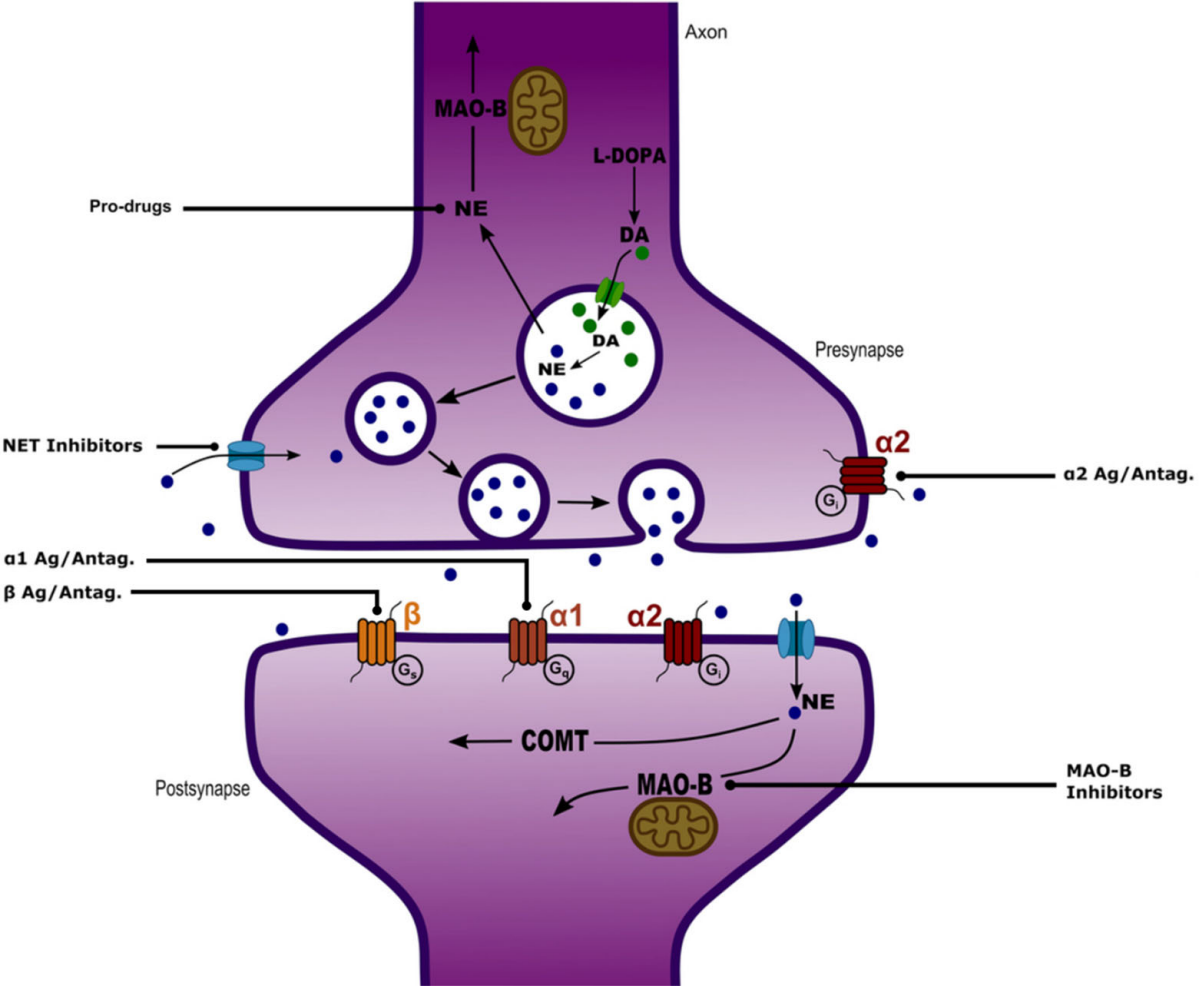
Schematic of a synapse showing the production of noradrenaline (from L‐DOPA) and its release across the synapse. Also, the reuptake and degradation of noradrenaline by COMT and MAO enzymes. Also shown are the key adrenergic receptors upon which it acts and the site of action of some key classes of drugs. DA, dopamine; NE, noradrenaline/norepinephrine; NET, norepinephrine transporter; L‐DOPA, levodopa; MAO, monoamine oxidase; COMT, catechol‐o‐methyltransferase.

Noradrenergic compounds can affect the LC‐NE system in various ways: by modifying the firing rate of the neurons, altering the release or reuptake of NE or by modulating stimulatory or inhibitory inputs to the LC^67^ (Fig. 5).

The majority of clinical trials investigating noradrenergic drugs in AD have been focused on improving the behavioural disturbances, such as aggression, that are often seen in the latter stages of the disease.^121^, ^122^ This does not preclude the possibility that noradrenergic stimulation might be of cognitive benefit when targeting specific subgroups of patients at an earlier stage of the disease process. The majority of evidence regarding the use of noradrenergic therapies in AD are from small‐scale, experimental trials that took place during several decades ago––at a time when the diagnostic criteria for AD were less clear.^8^, ^9^, ^10^ On the whole, these did not show positive effects, but there is renewed hope from more recent work.

### Guanfacine

Noradrenergic influence on PFC function seems especially pertinent in terms of augmenting cognition. This has been investigated extensively by Arnsten and colleagues, who have shown that guanfacine, an α_2A_‐AR agonist that acts primarily on post‐synaptic receptors in the PFC, can improve working memory, and top‐down control of attention across species.^25^

Guanfacine is a drug licenced for treatment of childhood ADHD that acts primarily on the PFC. Guanfacine enhances cognitive processes associated with the PFC including working memory, and top‐down control of attention, in healthy subjects as well as in patients with ADHD, autism, delirium, schizophrenia, substance abuse and post‐traumatic stress disorder.^25^ Several small studies looking at the effects of guanfacine in AD patients approximately 30 years ago found no effect.^9^, ^10^, ^123^ However the results of one subsequent, unpublished, open label study of 22 AD patients appeared to demonstrate that, when used in combination with the cholinergic drug donepezil, cognitive improvement was seen (see (15)). Our group are currently undertaking a Phase 3 trial of guanfacine in addition to cholinergic therapy in AD (‘NorAD’) (https://clinicaltrials.gov/ct2/show/NCT03116126).^124^

### Norepinephrine reuptake inhibitors

Reuptake of NE from the synapse terminates its action.^67^ Drugs known to increase alertness, such as modafinil, amphetamine and cocaine, act by inhibiting reuptake, as do antidepressants such as reboxetine, venlafaxine and duloxetine.^67^ Methylphenidate, the mainstay of ADHD treatment, acts by inhibiting both NE and dopamine reuptake in the PFC to improve attention and memory^125^ and has been suggested as a potential treatment for attentional impairment and apathy in AD^60^, with a recent placebo‐controlled study showing that it does reduce the latter.^126^

Atomoxetine is by far the most extensively studied drug of this class. As well as its established benefits in ADHD, atomoxetine has been shown to boost executive function in PD and is both safe and tolerable.^127^ Mohs et al. carried out a phase II placebo‐controlled trial of 25–80 mg/day of atomoxetine added to ongoing cholinesterase‐inhibitor in 92 mild‐to‐moderate AD patients. Patients were treated for up to 6 months and the researchers found that atomoxetine was well tolerated but not effective in improving cognitive function.^128^ A recent study in MCI showed that whilst atomoxetine significantly reduced CSF Tau and pTau, there were no significant treatment effects on cognition and clinical outcomes.^129^

### Non‐pharmacological approaches

Afferent fibres in the vagus nerve synapse at the nucleus tractus solitarius, which in turn projects to the LC.^130^ Whilst historically posited as having a predominantly dopaminergic mechanism, there is a strong argument that noradrenergic mechanisms underlie the effects of vagus nerve stimulation (VNS).^131^ For example, activation of the vagus nerve has been shown to stimulate the LC to release NE in rats,^132^ although findings from rodent studies must be taken with caution. In a single‐blind sham‐controlled randomised crossover pilot study in healthy older individuals, transcutaneous VNS has been shown to boost memory performance when compared to a sham‐controlled condition.^133^ VNS has also been trialled in AD. A series of studies in 1990 applying non‐invasive transcutaneous electrical nerve stimulation showed a positive effect on cognition and behaviour was seen but the effects disappeared after cessation of stimulation.^134^ Given its non‐invasive nature, limited side effects and efficacy after only a single session, this technique holds potential as a non‐pharmacological noradrenergic therapy. However, further investigation is required, particularly with respect to understanding the exact effects of VNS on the LC, identifying optimal stimulation parameters and ascertaining potential duration of effect.^135^

## Conclusions

The LC‐NE system has consistently and reliably been shown to be dysfunctional in AD, causing an array of challenging and distressing symptoms that are not adequately alleviated by current therapies. Therapeutic modulation of the LC‐NE system has the potential to improve not only patients' cognition, but also the less‐reported symptoms of autonomic dysfunction and neuropsychiatric disturbance that many suffer from. However, there are no trials to‐date showing a clear effect of noradrenergic treatments in AD.

A series of underpowered trials in a poorly defined patient population approximately 30 years ago did not show efficacy for noradrenergic treatments in AD. Subsequent developments in neuroimaging, including neuromelanin‐sensitive sequences and functional approaches, have improved our ability to interrogate the integrity and function of the LC‐NE system at the individual level. Other measures of noradrenergic tone, such as pupillometry and EEG, have the potential to be used dynamically and at greater scale. The rationale for noradrenergic treatment in AD is strong and there are a range of treatments, pharmacological and non‐pharmacological, that are known to be safe, tolerable and effective in other patient groups. In order to gain further understanding and direct personalised treatment approaches, biomarkers should, where possible, be used to inform research and may eventually guide clinical practice.

## Conflict of Interest

PM is the lead investigator for the NIHR‐funded NorAD trial. Investigational medicinal product and placebo for this study are provided via a ‘drugs‐only’ grant from Takeda (formerly Shire pharmaceuticals). This work was supported by the National Institute for Health Research (NIHR) Biomedical Research Centre at Imperial College London. MD is a Clinical Research Training Fellow funded by the Medical Research Council (MRC) (Grant Ref: MR/W016095/1).
